# Sustainable Routes Toward Amino Acid‐Based Surfactants: Borrowing Hydrogen Catalysis and Beyond

**DOI:** 10.1002/cssc.71075

**Published:** 2026-10-15

**Authors:** Maximilian Fellert, Ben L. Feringa

**Affiliations:** ^1^ Stratingh Institute for Chemistry University of Groningen Groningen Netherlands; ^2^ NanoEnergieTechnikZentrum, Center for Nanointegration Duisburg‐Essen University of Duisburg‐Essen Duisburg Germany

**Keywords:** amino acid, borrowing hydrogen, catalysis, chemical surfactants, organic chemistry

## Abstract

Amino acid‐based surfactants combine renewable head groups with tunable amphiphilic functional groups and are therefore attractive candidates for the development of more sustainable surfactants. However, bio‐based building blocks alone cannot guarantee sustainability. Moreover, it depends on the choice of synthetic route, waste generation, feedstock origin, catalyst, product performance, toxicity, and environmental fate. This perspective discusses borrowing hydrogen catalysis (BHC) as a central strategy toward *N*‐alkylated and *N*,*N‐*dialkylated amino acid‐based surfactants, which are derived from alcohols and amines with water as the only formal by‐product. To place BHC in context, complementary routes such as enzymatic *N*‐acylation, pathway‐engineered fermentation, and Diels–Alder/ring‐opening strategies starting from renewable hydrophobes are highlighted. Finally, design criteria and future challenges for the translation of amino acid‐based surfactants from promising lab studies toward scalable and sustainable platforms are proposed.

## Graphical abstract

This perspective examines how amino acid‐based surfactants become more sustainable only when molecular architecture is matched to an appropriate synthetic disconnection. Borrowing Hydrogen catalysis provides a central route to *N*‐alkylated derivatives, while enzymatic, biosynthetic, and renewable‐platform strategies complement it. Transparent metrics and process‐aware design guide translation toward scalable surfactant production.

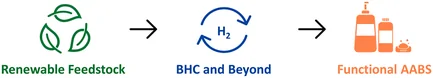

## 1 Introduction: Why Sustainable Synthesis Matters for Amino Acid‐Based Surfactants

Surfactants have become a part of our daily life and are consumed in large quantities worldwide in view of their widespread use in personal care products, in particular cleaning agents, soaps, and shampoos. Therefore, it is of general interest and importance to develop surfactants that are non‐toxic, consumer‐friendly, and cause less irritation [1, 2, 3].

Additionally, the need for renewable resources as feedstock chemicals for producing surfactants and enhancement of their biodegradability and biocompatibility has risen in the chemical industry’s major goals to use Green Chemistry as a contribution to an overall greater global sustainability and circular economy [4, 5, 6, 7, 8, 9, 10, 11, 12, 13, 14, 15, 16, 17, 18, 19, 20, 21, 22, 23, 24, 25].

Highly promising perspectives are offered by the use of amino acids as bio‐based starting materials for the synthesis and formulation of novel surfactants, which relies on the possibility of obtaining the hydrophilic part, i.e., the amino acid is obtained by fermentation [26] or extraction from protein hydrolysates [27] and the hydrophobic part is available from natural oils [28].

The interest in amino acid‐based surfactants can be traced back to 1909, when *N*‐acylated glycine and alanine were reported for the first time [29]. The first patent using the amino acid‐based surfactants acyl sarcosinate and acyl aspartate was filed in 1936 [30]. Major challenges remain, especially toward sustainable synthesis, despite the fact that amino acid‐based surfactants have been widely explored and found applications in various industries such as pharmaceuticals, cosmetics, and food chemistry [20].

## 2 Scope

This perspective is not aimed at providing a comprehensive review of all amino acid‐based surfactants and their applications, as recent reviews cover the whole breadth of the field, including synthesis, self‐assembly, foaming, rheology, antimicrobial activity, and their formulation [4, 5, 20, 31]. Instead, the focus is narrower and deliberately aligned with Green Chemistry. Selected synthetic pathways are compared according to how they are able to improve the sustainability profile of amino acid‐based surfactants. Thereby, borrowing hydrogen catalysis is treated as the central example because it enables *N*‐alkylation and *N*,*N*‐dialkylation using alcohols as alkylating agents with water as the only by‐product [12, 14, 32, 33]. To position this strategy properly, selected complementary green approaches are discussed as well, which include enzymatic *N*‐acylation, pathway‐engineered fermentation, and Diels–Alder/ring‐opening chemistry [16, 17, 18, 19].

Borrowing hydrogen catalysis is a powerful, yet not universal strategy to obtain amino acid‐based surfactants that is most directly applicable when the hydrophobic group is connected to the amino acid by C─N bond formation. By contrast, *N*‐acyl amino acids, glycerolipid‐like structures, and many gemini surfactants are more naturally accessed through acylation, esterification, amidation, or biocatalytic condensation [6, 16, 17, 34, 35]. Borrowing hydrogen catalysis can still contribute indirectly by preparing *N*‐alkylated amino acid building blocks or enabling late‐stage modification, but this role should be distinguished from the main bond‐forming step that creates glycerolipid or gemini frameworks [12, 13, 34, 35].

## 3 Structural Classes and Synthetic Disconnections

The structural diversity of amino acid‐based surfactants (AABS) requires different synthetic disconnections, and the sustainability problem is therefore not the same for every AABS class. Key information regarding main structural disconnection, classical synthetic route, arising sustainability issues, and alternative green routes is summarized in Table 1.

**TABLE 1:** Structural classes, synthetic disconnections, sustainability issues, and green alternatives for selected AABS.

AABS class	Structural motif (AA = amino acid)	Main structural disconnection	Classical route and sustainability issue	Greener route to discuss
*N*‐Acylated AABS	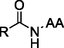	*N*‐Acylation of the amino group; usually amide bond formation.	Long‐chain acyl chlorides in Schotten–Baumann reactions; stoichiometric HCl and neutralization.	Enzymatic *N*‐acylation, fatty acid methyl esters, triglycerides, aminoacylases.
*N*‐Alkylated AABS	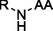	C─N bond formation at amino nitrogen.	Alkyl halides or reductive amination with stoichiometric reductants.	BHC using fatty alcohols; water as formal by‐product.
*N*,*N*‐Dialkylated AABS	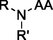	Double C─N bond formation at amino nitrogen.	Sequential alkylation, halide salts, or protecting‐group strategies.	Earth abundant metal‐catalyzed alcohol coupling; Shvo‐type systems.
Betaine‐like or amphoteric AABS	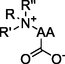	Quaternary ammonium/carboxylate structures.	Quaternization with alkyl halides and salt generation.	BHC‐derived *N*‐alkyl precursors, then functionalization where needed.
Gemini/dimeric AABS	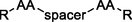	Two amphiphilic units connected by spacer.	Multi‐step coupling/quaternization; protecting groups; halogenated spacers.	Platform routes, enzymatic coupling, lysine bi‐acylation, or indirect BHC building blocks.
Glycerolipid‐like AABS	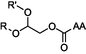	Amino acid head and hydrophobic chains connected through glycerol core.	Chemical esterification/acylation, often protecting‐group dependent.	Lipase/protease‐mediated condensation and step‐economic esterification.
Fermentative acyl amino acids	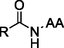	Biosynthetic assembly of acyl amino acid products.	Classical surfactant production from petrochemical or seed‐oil feedstocks.	NRPS/pathway engineering and cellulosic feedstocks.

*N*‐Acylated AABS are primarily obtained through *N*‐acylation of the amino group, resulting in amide bond formation; industrially relevant routes often rely on Schotten–Baumann acylation with long‐chain acyl chlorides, which is effective but uses reactive chlorine chemistry and generates acid or salt waste upon neutralization. Greener alternatives include enzymatic *N*‍‐‍acylation, aminoacylase‐mediated synthesis, and the replacement of acyl chlorides by fatty acid methyl esters or triglycerides, which reduces the reliance on corrosive acyl chloride chemistry [4, 16, 17, 36].

*N*‐alkylated and *N*,*N*‐dialkylated AABS, by contrast, are most directly associated with C─N bond formation at the amino nitrogen. Classical approaches to such structures often involve alkyl halides, reductive amination, sequential alkylation steps, halide salts, stoichiometric reductants, or protecting‐group strategies. Borrowing hydrogen catalysis offers a more direct disconnection because amino acids, amino acid esters, or amino acid amides can be coupled with alcohols, including fatty alcohols, with water as the formal by‐product. For *N*,*N*‐dialkylated structures, this logic can be extended to double C─N bond formation at nitrogen, with Ru‐catalyzed, Shvo‐type, and Ir‐catalyzed alcohol‐coupling strategies providing the most relevant literature precedents [12, 13, 14, 37].

Betaine‐like or amphoteric AABS introduce an additional design element because their structures combine ammonium or carboxylate functionality with an amphiphilic character. Conventional access to quaternary ammonium‐type amino acid surfactants commonly involves quaternization with alkyl halides and therefore creates salt‐containing product streams. In a sustainability‐oriented framework, BHC‐derived *N*‐alkyl amino acid precursors can be used as upstream building blocks before targeted functionalization, although any final quaternization or zwitterion‐forming step must still be evaluated separately [12, 38, 39].

Gemini and dimeric AABS are structurally distinct because they contain two amphiphilic units connected by a spacer. Classical access to such structures often requires multi‐step coupling, quaternization, protecting group manipulations, or halogenated spacers. More sustainable alternatives include enzymatic coupling strategies, platform routes that allow spacer and head‐group variation, lysine‐based bi‐acylation, and the indirect use of BHC‐derived amino acid building blocks. Valivety and coworkers demonstrated enzyme‐assisted routes to amino acid‐based bola and gemini surfactants, while Jolmes and coworkers showed that lysine‐containing platform chemistry can provide either mono‐acylated derivatives or bi‐acylated gemini‐type surfactants [6, 12, 19, 35].

Glycerolipid‐like AABS are best understood as amino‐acid head‐group/hydrophobic‐chain conjugates connected through a glycerol linker. Their synthesis is therefore more naturally associated with esterification or acylation chemistry than with BHC as the main framework‐forming reaction. Chemical esterification and acylation can require protecting‐group‐dependent sequences, whereas lipase‐ or protease‐mediated condensation provides a greener and more step‐economic route to glycero amino acid‐based surfactants and amino acid glyceride conjugates [34, 35, 40].

Fermentative acyl‐amino acid‐based surfactants represent a separate category because their key structural motif is assembled biosynthetically rather than through classical small‐molecule coupling chemistry. This route addresses a different sustainability issue: conventional surfactant production often depends on petrochemical or seed‐oil feedstocks, whereas engineered biosynthetic pathways can use renewable carbohydrate streams. Reznik and coworkers engineered the native surfactin synthetase pathway in *Bacillus subtilis* to produce an acyl amino acid surfactant and discussed cellulosic carbohydrate feedstocks as a route toward more sustainable surfactant manufacturing. This example should be presented as complementary to BHC, because it changes the feedstock and production logic rather than merely replacing one chemical bond‐forming reagent with another [10, 18, 20].

Having mapped the structural classes of AABS to their most appropriate sustainable disconnections, the following section focuses on Borrowing Hydrogen catalysis as the central strategy for alcohol‐based C─N bond formation in *N*‐alkylated and *N*,*N*‐dialkylated amino acid‐derived surfactants.

## 4 Borrowing Hydrogen Catalysis (BHC) as a Central Route to *N*‐Alkylated AABS

The Borrowing Hydrogen strategy relies on the combination of a transfer hydrogenation that enables the avoidance of the direct use of H_2_, with at least one additional intermediate reaction to obtain complex molecules without the effort of tedious separation or isolation processes [41]. Thereby, hydrogen, coming from a donor molecule, is transferred to a catalytic metal fragment, i.e., the “borrowing” of H_2_ is carried out in a final hydrogenation step. The single steps of the mechanism for the catalytic coupling of both building blocks, producing only water as a waste, can be illustrated using the example of amine *N*‐alkylation (Scheme 1) [32].

**SCHEME 1:**
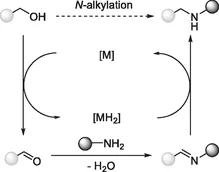
Generalized transition‐metal‐catalyzed *N*‐alkylation of an alcohol using the BH methodology. Adapted with permission [32]. Copyright 2025, Wiley‐VCH GmbH.

The process is initiated by the transition‐metal‐catalyzed dehydrogenation of the alcohol, forming a reactive carbonyl intermediate. The hydrogen that was abstracted from the alcohol was temporarily transferred to the transition‐metal catalyst and stored there. The reactive carbonyl species subsequently undergoes condensation with an amine, forming an imine. The final step involves the reduction of the previously formed imine using the hydrogen that was borrowed from the alcohol donor in the first step and is released from the transition‐metal catalyst. This sequence of reaction steps comes with a variety of advantages, i.e., producing water as the sole by‐product, high atom economy, and thus favorable E‐factor metrics, making BH an important methodology for a transition to a greener chemical future.

Over the past years, due to its potential to contribute to a greener way of performing chemical reactions, the BH methodology has gained more and more attention [32, 33, 42]. Both homogenous and heterogenous variants using a broad spectrum of catalysts, either based on transition metals or more earth‐abundant metals such as iron and manganese, have been developed and employed in different chemical transformations. For the *N*‐alkylation of aliphatic alcohols with aliphatic amines, the type of reaction we focused on in recent years, a variety of catalysts have been developed, being based on either ruthenium [13, 41, 43, 44, 45, 46, 47, 48, 49], iridium [50, 51, 52], nickel [53], or cobalt [54].

### 4.1 Borrowing Hydrogen Catalysis‐Derived Amino Acid‐Based Surfactants: A Proof‐of‐Concept Study

Our proof‐of‐concept synthesis of bio‐based surfactants via borrowing hydrogen catalysis demonstrates that this disconnection can be translated from small‐molecule amine synthesis to functional amphiphiles. We combined ubiquitous amino acids and common alcohols without protecting‐group manipulations and synthesized amino acid‐derived surfactants in a single step using a commercially available ruthenium catalyst; in the same study, we further showed that BHC‐derived products can be converted into Gemini surfactants and a related quaternary ammonium salt (Figure 1) [12].

**FIGURE 1:**
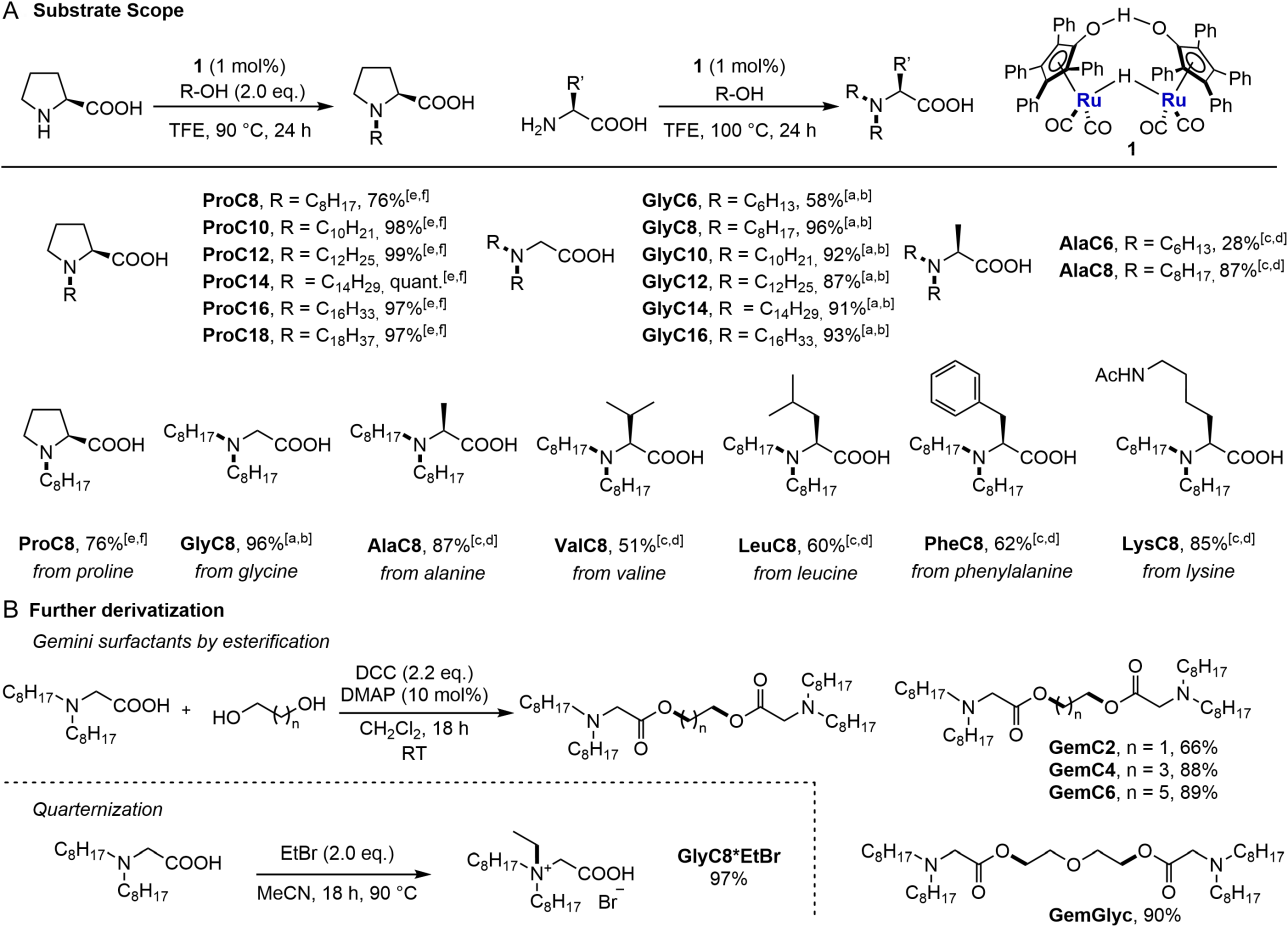
Substrate scope and further derivatization of the surfactants. ^a^Small scale: glycine (1.0 equiv.), alcohol (2.4 equiv.), 1 (1 mol%), TFE (0.2 M), 100 °C, 24 h. ^b^Large scale: glycine (1.0 equiv.), alcohol (3.0 equiv.), 1 (0.5 mol%), TFE (1.0 M), 100 °C, 24 h. ^c^Small scale: amino acid (1.0 equiv.), alcohol (4.0 equiv.), 1 (1 mol%), TFE (0.2 M), 100 °C, 24 h. ^d^Large scale: amino acid (1.0 equiv.), alcohol (4.0 equiv.), 1 (0.5 mol%), TFE (1.0 M), 100 °C, 24 h. ^e^Small scale: proline (1.0 equiv.), alcohol (2.0 equiv.), 1 (1 mol%), TFE (0.2 M), 90 °C, 24 h. ^f^Large scale: proline (1.0 equiv.), alcohol (1.2 equiv.), 1 (0.5 mol%), TFE (1.0 M), 90 °C, 24 h. Reproduced with permission [12]. Copyright 2025, Wiley‐VCH GmbH.

Compared with classical *N*‐alkylation using alkyl halides or alkyl sulfonates, BHC can avoid stoichiometric salt formation and selectivity problems; compared with reductive amination, it avoids the need for preformed aldehydes and external stoichiometric hydride donors [13, 14, 42, 55, 56].

Earlier studies by our group established that unprotected amino acids as well as α‐amino acid esters and amides can undergo direct catalytic *N*‐alkylation with alcohols, providing the synthetic foundation for the later AABS proof‐of‐concept [13, 14].

From the surfactant perspective, the key lesson is that BHC‍‐‍derived AABS must be evaluated not only by yield and step count but also by structure‐dependent properties such as surface tension reduction, CMC, foaming behavior, solubility, and biodegradability. These properties are known to depend strongly on the head‐group structure, hydrophobic tail, substitution pattern, and interfacial packing [12, 57, 58].

Therefore, sustainability claims should distinguish between the intrinsic reaction economy of the BHC disconnection and the practical process footprint of the laboratory protocol. A complete evaluation requires explicit mass‐balance data for solvent, catalyst loading and recovery, work‐up, purification, salts, and isolated yield, ideally reported as an experimental E‐factor or process mass intensity in addition to an idealized stoichiometric E‐factor [8, 9, 32, 59].

### 4.2 Green Metrics: An E‐Factor Example for a BHC‐Derived AABS

The E‐factor is defined as the mass of waste generated per mass of the isolated product. Sheldon originally introduced it as a simple metric for pollution prevention, and later discussions emphasize that process E‐factors should account for solvents, work‐up materials, and auxiliary substances when the required data are available [60]. For AABS obtained by BHC, it is useful to distinguish an idealized reaction E‐factor from a complete process E‐factor. An example calculation is depicted in Table 2.

**TABLE 2:** Idealized E‐factor for a formal BHC synthesis of *N*,*N*‐dinonylglycine [12, 60].

Formal reaction: glycine + 2 equiv. 1‐nonanol → *N*,*N*‐dinonylglycine + 2 equiv. H_2_O.
Molecular masses: glycine 75.07 g mol^−1^; 1‐nonanol 144.26 g mol^−1^; *N*,*N*‐dinonylglycine 327.55 g mol^−1^; water 18.02 g mol^−1^.
Stoichiometric waste considered in the idealized reaction: 2 × H_2_O = 36.04 g mol^−1^.
Idealized reaction E‐factor = 36.04/327.55 = 0.11 kg waste per kg product.
Atom economy = 327.55/(75.07 + 2 × 144.26) = 90.0%.
Interpretation: this value represents only the intrinsic stoichiometry of the BHC disconnection. A complete process E‐factor would additionally require solvent masses, catalyst loss, work‐up reagents, salts, chromatographic materials, and purification solvents.

This calculation is intentionally conservative in its interpretation. It demonstrates the intrinsic atom economy of the BHC bond‐forming event, but it does not prove that a specific laboratory procedure already has a low process E‐factor. For future AABS studies, both values should be reported where possible: the idealized reaction E‐factor to compare disconnections and the experimental process E‐factor to compare practical protocols. This distinction is especially important when reactions are performed on a small scale and purified by chromatography [12, 60].

Thus, the idealized E‐factor should be read as a measure of the intrinsic potential of the BHC disconnection rather than as a full process metric; realizing this potential in scalable surfactant production will require targeted optimization of catalyst recovery, solvent management, feedstock use, and purification strategy.

### 4.3 Future Directions for BHC‐Derived AABS Synthesis

Having demonstrated that Borrowing Hydrogen catalysis can provide direct access to amino acid‐derived surfactants, the next step is to define the chemical and process‐level requirements that would allow this strategy to evolve from a proof‐of‐concept into a scalable platform for sustainable surfactant production (Figure 2).

**FIGURE 2:**
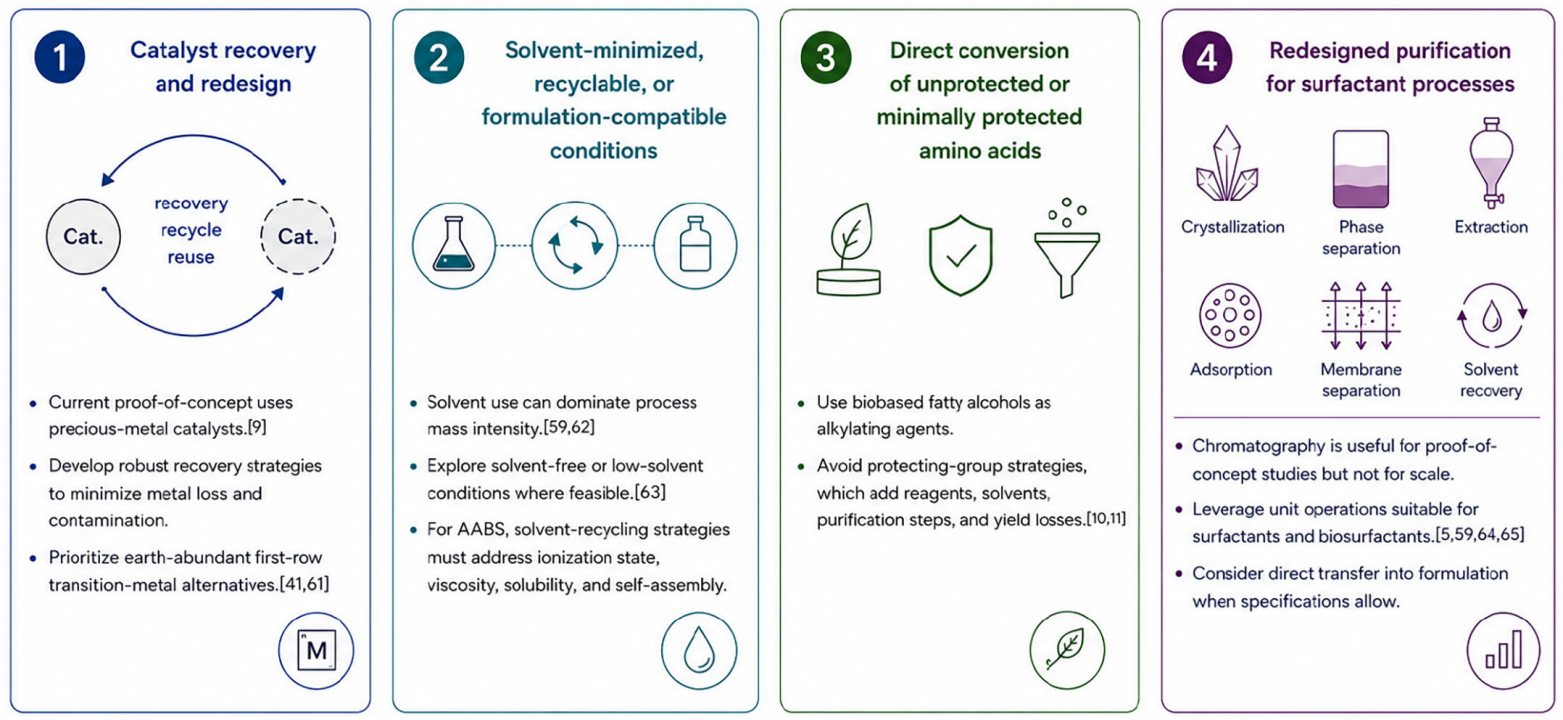
Future directions for BHC‐derived AABS synthesis.

At this stage, the key question is no longer only whether amino acid‐derived surfactants can be obtained by BHC, but whether the reaction can be translated into a process that remains attractive when catalyst supply, solvent demand, work‐up, purification, and product isolation are included in the sustainability assessment. This distinction is important because green metrics such as E‐factor and process mass intensity depend not only on the formal stoichiometry of the reaction but also on solvent use, auxiliary materials, yield, work‐up, and recovery operations [8, 9, 59].

The intrinsic appeal of BHC arises from its ability to use alcohols as alkylating agents and to form water as the formal stoichiometric by‐product [32, 43], but this formal mass efficiency must be distinguished from the practical process footprint, which also includes solvent use, catalyst loss, work‐up, purification, and isolated yield [9, 59].

First, catalyst recovery and catalyst redesign should become central development targets because the current proof‐of‐concept relies on precious‐metal catalysis [12], whereas broader BHC literature has shown increasing progress with earth‐abundant first‐row transition‐metal catalysts [42, 61].

Second, reactions should be optimized under solvent‐minimized, solvent‐recyclable, or formulation‐compatible conditions since solvent use can dominate process mass intensity even when the formal reaction stoichiometry is attractive [59, 62]. Solvent‐free BHC has already been demonstrated for selected amine syntheses [63], but amino acid‐derived surfactants may require more pragmatic solvent‐recycling strategies because the ionization state, viscosity, solubility, and self‐assembly can complicate mass transfer.

Third, direct conversion of unprotected or minimally protected amino acids with biobased fatty alcohols should be prioritized because protecting‐group strategies introduce additional reagents, solvents, purification steps, and yield losses [13, 14].

Fourth, purification should be redesigned for surfactant processes rather than copied from small‐molecule discovery chemistry: chromatography is useful for proof‐of‐concept studies, but scalable surfactant production should preferentially consider crystallization, phase separation, extraction, adsorption, membrane separation, solvent recovery, or direct formulation where product specifications allow [8, 59]. Membrane‐based downstream processing has already been explored for biosurfactant recovery and purification [64, 65], providing useful analogies for future BHC‐derived AABS processes.

## 5 Green Synthetic Strategies Toward AABS Beyond BHC

While BHC provides a particularly direct and atom‐economic route to *N*‐alkylated and *N*,*N*‐dialkylated AABS, it cannot address the full structural diversity of amino acid‐based surfactants. Complementary green synthetic strategies are therefore required for AABS classes whose defining motifs are formed through acylation, esterification, biocatalytic condensation, or biosynthetic assembly. The following sections discuss three representative examples: enzymatic *N*‐acylation as a more sustainable route to commercially relevant *N*‐acyl AABS, pathway‐engineered fermentation as a biotechnological strategy for acyl amino acid surfactants from renewable carbohydrate feedstocks, and Diels–Alder/ring‐opening chemistry as a platform approach that combines renewable hydrophobes with amino acid head groups (Figure 3).

**FIGURE 3:**
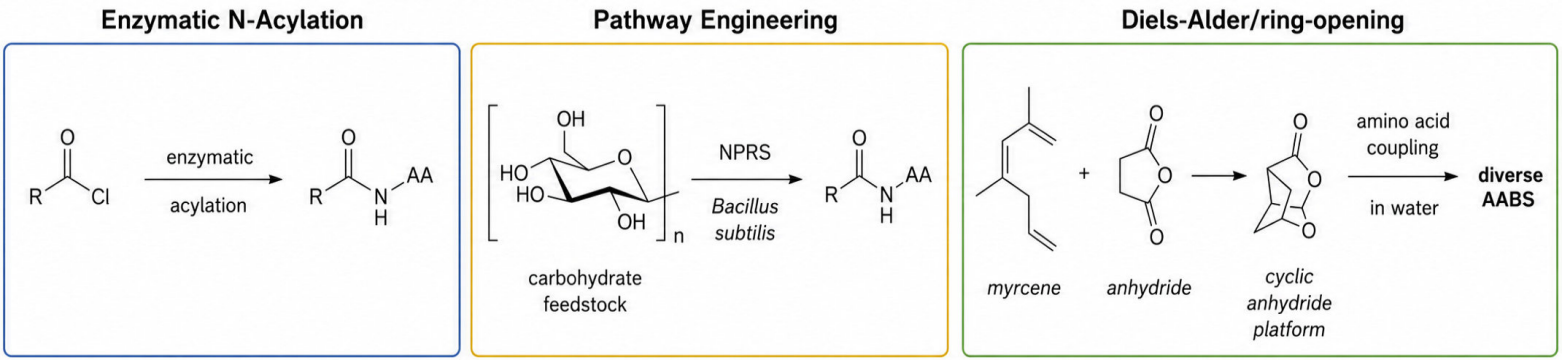
Green synthetic strategies toward AABS beyond BHC.

### 5.1 Enzymatic *N*‐Acylation for Commercially Relevant *N*‐Acyl AABS

As outlined previously, *N*‐acyl amino acid surfactants are an industrially important AABS class that is commonly accessed by Schotten–Baumann acylation with long‐chain acyl chlorides, a route associated with reactive chlorine chemistry and stoichiometric salt formation upon neutralization [5, 16, 17, 36]. Holmberg, Bilén, and Bordes therefore highlight that replacing acyl chlorides with triglycerides, fatty acid methyl esters, or enzyme‐compatible acyl donors is attractive from a toxicity and environmental perspective, while also noting that not all alternatives are equally green; DCC‐mediated coupling, for example, still relies on a stoichiometric coupling reagent [4, 9]. Recent enzymatic and chemo‐enzymatic studies support this direction: lipase‐catalyzed transacylation of palm oil fractions with amino acids has been explored as a route to medium‐ and long‐chain amino acid surfactants, aminoacylases from *Streptomyces ambofaciens* catalyze *N*‐acylation of L‐amino acids in an aqueous medium, and current reviews identify acylases, lipases, and esteramide interconversion cascades as promising routes to *N*‐acyl amino acid amides [66, 67, 68].

The recent enzymatic acylation of glycine by Kurnosov, Galatini, and Banfi is therefore a highly relevant example. The authors describe a chemoselective enzymatic route to *N*‐acyl amino acid surfactants under mild conditions and report high product formation from fatty acid esters and amino acid esters without relying on corrosive acid chlorides [16]. This example is particularly useful because it addresses a class that BHC does not access directly: whereas BHC is most powerful for *N*‐alkylated and *N*,*N*‍‐‍dialkylated architectures, enzymatic acylation directly targets the amide‐forming disconnection that defines *N*‐acyl AABS [13, 14, 16]. For *N*‐acyl AABS, the most relevant sustainability improvement is the replacement of acyl chloride chemistry by enzyme‐compatible acylation routes, such as aminoacylase‐mediated *N*‐acylation, lipase‐catalyzed transacylation, or ester‐based acyl transfer [17, 67].

Biocatalytic *N*‐acylation is, nevertheless, not yet a universal solution. Substrate scope, acyl donor compatibility, water activity, miscibility of fatty acyl donors and amino acid substrates, enzyme stability, product inhibition, enzyme cost, and downstream processing remain important constraints [17, 68, 69]. Earlier chemo‐enzymatic work already showed that low miscibility of reactants can limit yields when free amino acids or their derivatives are acylated with fatty acids, whereas substrate protection or linker strategies can improve conversion but may reduce the overall green advantage [69]. Thus, enzymatic *N*‐acylation should be discussed alongside BHC as a complementary green strategy: it does not replace BHC for *N*‐alkylated AABS, but it directly addresses the sustainability limitation of the industrially important *N*‐acyl family [16, 17].

### 5.2 Biotechnological Production Through Pathway Engineering

A second route beyond small‐molecule catalysis is direct biotechnological production. Reznik and coworkers engineered the native nonribosomal peptide synthetase machinery responsible for surfactin biosynthesis in *Bacillus subtilis* to produce a fatty‐acyl glutamate derivative rather than the parent lipopeptide surfactin [18]. The study is instructive because it converts a complex biosurfactant concept into a simpler acyl amino acid surfactant and links production to carbohydrate feedstocks of lignocellulosic origin. More broadly, surfactin biosynthesis is carried out by nonribosomal peptide synthetase systems in *Bacillus* strains, and recent work on microbial biosurfactant production shows that strain engineering, feedstock selection, and process design are central bottlenecks for translating biosurfactants into industrially relevant products [70, 71].

The performance comparison in the Reznik study is also useful for reframing the numerical surfactant data. FA‐Glu was soluble up to 312 mM, whereas myristoyl glutamate was soluble up to 89 mM, and FA‐Glu showed a substantially lower CMC than myristoyl glutamate, 1.3 mM versus 14.1 mM. Importantly, the biosynthetically produced FA‐Glu was not a single molecular species but a mixture of β‐hydroxy fatty‐acyl glutamates differing in chain length and branching, with C14 species accounting for approximately 70% of the purified product [18]. The markedly higher aqueous solubility of FA‐Glu can therefore be rationalized, at least in part, by the additional β‐hydroxyl group in the fatty‐acyl moiety, which increases the polarity and hydration capacity of the hydrophobic domain compared with that of myristoyl glutamate. The lower CMC is likewise consistent with the strong dependence of amino acid‐based surfactant self‐assembly on hydrophobic‐chain length, branching, functionality, and molecular packing [58]. However, because FA‐Glu represents a mixture of molecular species, the approximately tenfold difference in CMC should not be attributed to a single structural parameter. These values should not be presented as isolated property data; instead, they support a sustainability‐relevant interpretation. Higher aqueous solubility can reduce formulation constraints, and lower CMC values can reduce the amount of surfactant required to reach micellar or interfacial activity, although the final dose advantage always depends on the target application and formulation matrix [18, 57, 58].

The production data further show the relevance of the feedstock choice. FA‐Glu was produced from soybean hulls and purified cellulose‐derived carbohydrates such as cellobiose and xylose, and the reported experiments showed that cellobiose and xylose as sole carbon sources gave higher FA‐Glu titers than glucose. This finding is consistent with broader biosurfactant research, in which agro‐industrial residues, crude glycerol, rapeseed cake, sunflower cake, and other secondary feedstocks are being investigated to reduce the cost and environmental burden of microbial surfactant production [70, 71]. This example complements BHC: whereas BHC improves the chemical disconnection and avoids preactivated alkylating agents, pathway engineering can integrate renewable feedstocks, microbial conversion, and product formation into a single production platform [10, 18].

### 5.3 Diels–Alder/Ring‐Opening Chemistry From Renewable Hydrophobes

Diels–Alder/ring‐opening chemistry provides a third route to bio‐based AABS that does not rely on fatty acid chlorides. Jolmes and coworkers used the monoterpene myrcene as a renewable hydrophobic building block and reacted it with maleic or citraconic anhydride to generate cyclic anhydride precursors [19]. Myrcene is particularly suitable for such platform chemistry because it is an industrially available terpene‐derived diene and has been discussed as a renewable base chemical for sustainable chemistry [72]. In related work, Hornung and coworkers demonstrated Diels–Alder reactions of myrcene with various dienophiles under intensified continuous‐flow conditions, supporting the broader idea that myrcene‐based cycloaddition chemistry can be developed beyond small‐batch reactions [73].

In the Jolmes study, subsequent ring opening of the cyclic anhydrides with amino acids in an aqueous medium furnished a structurally diverse set of bio‐based anionic and amphoteric surfactants. The route is attractive from a green chemistry perspective because the cycloaddition has high atom economy and avoids stoichiometric coupling reagents; for citraconic anhydride, the liquid substrate also enabled a solvent‐free Diels–Alder step under the reported microwave conditions [9, 19, 74]. This route therefore addresses a different sustainability problem than BHC: instead of replacing alkyl halides for *N*‐alkylation, it creates an anhydride platform from a renewable hydrophobe and then couples this platform to amino acid head groups under aqueous conditions [19, 74].

The platform character is important. By varying the amino acid, Jolmes and coworkers accessed different charge states and performance profiles, and lysine gave access to mono‐acylated derivatives as well as a bi‐acylated gemini‐type surfactant. This illustrates how one renewable hydrophobe platform can generate a family of AABS without using acyl chloride chemistry while also connecting the synthetic route to molecular architecture [6, 19]. The same study provides property data that should be used selectively rather than as a general application excursus. *N‍*‐‍(myrcene‐MA)‐serine and *N*‐(myrcene‐MA)‐tryptophan showed CMC values of approximately 2 and 3 mmol L^−1^, respectively, and at pH 5.5, polar amino acid derivatives such as arginine, serine, tryptophan, and lysine showed high foam heights and improved foam stability. These observations are relevant to the present perspective because formulation performance can influence the applied dose and because the route links renewable hydrophobes, amino acid head groups, and application‐relevant properties in a single platform concept [4, 19].

## 6 Design Rules for Sustainable AABS Synthesis and Process Development

The comparison of BHC with enzymatic acylation, biotechnological production, and renewable hydrophobe platform chemistry highlights that sustainable AABS development is best understood as a structure‐specific design problem rather than as a one‐route‐fits‐all challenge. The following design rules summarize the resulting principles for future synthesis‐ and process‐oriented studies (Figure 4).

**FIGURE 4:**
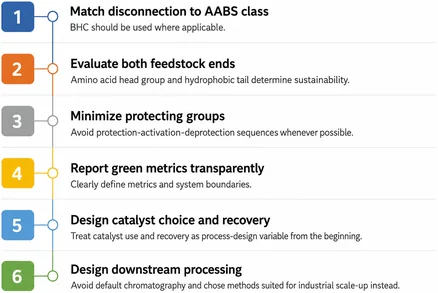
Design rules for sustainable AABS synthesis and process development.

First, the synthetic disconnection should match the structural class. BHC should be used where the defining bond‐forming step is *N*‐alkylation or *N*,*N*‐dialkylation with alcohols, whereas *N*‐acylated surfactants are more appropriately addressed through enzymatic acylation, aminoacylase‐mediated synthesis, fatty acid methyl ester routes, triglycerides, or related acyl‐donor strategies [12, 13, 14, 16, 17].

Second, renewable feedstocks should be evaluated at both ends of the molecule. Amino acids provide renewable and structurally tunable polar building blocks, but the hydrophobic component and its origin are equally important. Fatty alcohols, fatty acid methyl esters, triglycerides, terpene‐derived platforms such as myrcene, and carbohydrate feedstocks for fermentation address different parts of the feedstock challenge and should therefore be discussed as complementary rather than interchangeable solutions [4, 10, 12, 18, 19]. This rule is important because a bio‐based head group alone does not define a sustainable surfactant platform; the hydrophobic residue, production route, and feedstock supply chain must also be considered.

Third, protecting group‐minimized synthesis should be prioritized. Each protection, activation, deprotection, and intermediate purification step introduces additional reagents, solvents, waste streams, and yield losses, which can erode the green advantage of renewable starting materials. Direct BHC of unprotected amino acids or minimally protected amino acid derivatives with alcohols is therefore particularly attractive for *N*‐alkylated AABS, whereas enzymatic and chemoenzymatic routes are preferable when the target structure requires acyl transfer rather than *N*‐alkylation [12, 13, 14, 16].

Fourth, green metrics should be reported transparently and with clearly defined system boundaries. Atom economy and idealized stoichiometric E‐factors are useful for comparing reaction concepts, but they should not be confused with process E‐factors or process mass intensity. A reaction that formally produces only water can still have a substantial practical footprint if it requires large solvent volumes, high catalyst loading, extensive work‐up, chromatographic purification, or low isolated yields [8, 9, 59]. Sheldon defines the E‐factor as a central waste metric in green chemistry, while process mass intensity is commonly used to capture broader material demand in process development [8, 9].

Fifth, catalyst choice and catalyst recovery should be treated as process‐design variables from the beginning. Our current BHC proof‐of‐concept for AABS relies on precious‐metal catalysis, which is appropriate for demonstrating feasibility, but questions of catalyst recovery, metal loss, cost, supply risk, or product contamination still need to be evaluated in more detail [12]. Future studies should therefore evaluate recoverable catalyst systems and, where possible, earth‐abundant first‐row transition‐metal alternatives while recognizing that amino acid‐derived surfactants may impose additional challenges through polarity, coordination ability, ionization state, and product aggregation. Recent BHC reviews document the growing role of first‐row transition metals and commodity alcohols in hydrogen‐autotransfer chemistry [32, 42, 61].

Sixth, purification and downstream processing should be designed for surfactants rather than copied from small‐molecule discovery chemistry. Chromatography may be acceptable for proof‐of‐concept studies, but scalable AABS production should prioritize crystallization, phase separation, extraction, adsorption, membrane separation, solvent recovery, or direct transfer into formulation‐compatible matrices, where product specifications allow. This point is especially important because purification can dominate solvent demand and process mass intensity even when the formal reaction stoichiometry is attractive [8, 59, 64, 65].

## 7 Conclusions

Amino acid‐based surfactants offer a versatile platform for more sustainable surfactant chemistry, but their sustainability must be designed rather than assumed. Their molecular appeal arises from the combination of amino acid‐derived polar groups with tunable hydrophobic residues, yet the overall sustainability profile depends on the full synthetic and process context, including feedstock origin, bond‐forming strategy, catalyst system, waste generation, solvent demand, purification, and scalability [4, 9, 10].

Borrowing hydrogen catalysis provides a particularly attractive route to *N*‐alkylated and *N*,*N*‐dialkylated amino acid‐based surfactants because it enables C─N bond formation from alcohols with water as the formal stoichiometric by‐product [12, 13, 14]. Its strongest role is therefore not as a universal solution for all AABS architectures but as a structure‐specific disconnection for surfactants in which the hydrophobic residue is introduced through *N*‐alkylation. Other important AABS families require complementary strategies: enzymatic *N*‐acylation addresses commercially relevant *N*‐acyl surfactants, pathway engineering links surfactant production to renewable carbohydrate feedstocks, and Diels–Alder/ring‐opening chemistry demonstrates how renewable hydrophobes can be coupled with amino acid head groups in platform approaches [16, 18, 19].

Future progress will depend on translating elegant synthetic disconnections into practical surfactant processes. For BHC‐derived AABS, this means moving beyond formal atom economy toward transparent process metrics that include solvent use, catalyst loading and recovery, work‐up, purification, and isolated yield [8, 59]. The most promising developments will combine renewable amino acid and hydrophobic feedstocks with protecting‐group‐minimized synthesis, recoverable or earth‐abundant catalysts, solvent‐conscious reaction design, and downstream processing strategies suited to surfactant manufacture. In this broader framework, BHC is a powerful component of sustainable AABS chemistry, especially for *N*‐alkylated derivatives.

## Conflicts of Interest

The authors declare no conflicts of interest.

## Data Availability

Data sharing is not applicable to this article as no datasets were generated or analyzed during the current study.
